# Duration of EEG monitoring needed to ensure a low risk of seizure recurrence in hospitalized patients

**DOI:** 10.1007/s00415-025-13596-x

**Published:** 2026-01-09

**Authors:** Parimala Velpula Krishnamurthy, Santiago Philibert-Rosas, Maxwell Rivkin, Cameron J. Brace, Steven E. Haworth, Safoora Fatima, Atakan Selte, Mariel Kalkach Aparicio, Aaron F. Struck

**Affiliations:** 1 Department of Neurology, University of Wisconsin - Madison, Madison, WI USA; 2 Department of Neurology, Washington University in St. Louis, St. Louis, MO USA; 3 Department of Neurology, Southern Illinois University, Springfield, IL USA; 4 Department of Neurology, University of California Los Angeles – Harbor, Los Angeles, CA USA; 5 Department of Neurology, Sanford Medical Center, University of South Dakota, Sioux Falls, SD USA

**Keywords:** Continious electroencephalogram (cEEG), Seizure recurrance, Status epilepticus, EEG monitoring

## Abstract

**Background:**

Continuous EEG (cEEG) monitoring is critical for detecting seizures in hospitalized patients, but optimal durations of EEG monitoring remain unclear, particularly after a seizure has been detected on monitoring.

**Methods:**

We conducted survival analysis on 117 patients with electrographic or electroclinical seizures who underwent cEEG at the University of Wisconsin Hospital (UWH) between 2018 and 2022. Time from the end of seizure-to-seizure recurrence or cEEG termination was analyzed using Cox regression.

**Results:**

In univariate analysis, status epilepticus (SE) was the only clinical feature significantly associated with increased risk of seizure recurrence (*P* = 0.022). The estimated EEG duration required to reduce seizure recurrence risk below 5% was 36.8 h in patients with SE and 21.2 h in those without SE. Numerous other clinical variables, including coma, antiseizure medications addition, use of anesthetic infusions, history of epilepsy, and epileptiform discharges, were not significant.

**Conclusion:**

Our findings support current clinical practices of at least 24 h of EEG monitoring following seizure cessation and highlight that patients with a history of status epilepticus may require longer monitoring. These data reinforce the value of individualized, risk-based approaches to EEG monitoring strategies.

## Background

First developed in 1924 by German scientist Hans Berger [1, 2], electroencephalography (EEG) has been the gold standard in assessing cerebral electrical activity for the last one hundred years [3]. While commonly used in the management and identification of a wide variety of neurological perturbations, such as encephalopathies and acute brain injuries [4, 5], EEG is primarily used in clinical practice to detect seizures and seizure-related patterns [6].

Traditional EEG is valuable for seizure and pattern detection but faces practical barriers, including cost and setup time, limiting its use in emergent and critical care. The introduction of digital EEG in the 1990s expanded ICU monitoring, though many of these constraints persist [7]. Despite its pitfalls, the implementation of digital EEG has resulted in critical findings related to the field of epilepsy and seizure study. Primarily that seizures are more common than anticipated within the hospitalized population. As well as confirming that seizures are not solely seen in patient with acute brain injuries but rather can be seen across a wide variety of conditions ranging from local injuries such as subarachnoid hemorrhages to systemic conditions such as sepsis [3, 4, 8, 9].

Among critically ill adults undergoing continuous EEG monitoring, electrographic seizures are detected in approximately 12–27% of patients and most lack an overt clinical correlate [3, 10, 11]. In selected ICU cohorts, nonconvulsive status epilepticus (NCSE) prevalence has been reported in a minority to nearly half of patients undergoing cEEG monitoring, reflecting differences in patient selection and monitoring indications. Continuous EEG is therefore useful not only for seizure detection, but also for identifying EEG features associated with higher seizure risk and guiding monitoring duration [12].

There have been significant efforts to determine how long a patient needs to be on an EEG to ensure the seizure risk is low [11, 13–15]. Current recommendations are that at least 24 h of seizure freedom is required prior to cessation of EEG [16], but questions remain. Do the number of seizures, use of anti-seizure medications, anesthetic infusion, seizure burden, or duration of seizures influence the likelihood of seizure recurrence and the duration required to ensure low seizure risk? Are there clinical factors, such as underlying etiology or medications, that influence the necessary EEG duration to ensure low risk of seizure? In this study, we aim to add to the corpus of knowledge surrounding these questions.

## Methods

### Study design

We identified 117 patients who underwent continuous EEG (cEEG) monitoring at University Health Hospital in Madison, Wisconsin, and had an electrographic or electroclinical seizure during their monitoring. Patients undergoing cEEG within the epilepsy monitoring unit were excluded. This study received approval from the University of Wisconsin—Madison Institutional Review Board (IRB), with a waiver of consent granted.

### Patient selection

All patients admitted to University Health Hospital who received cEEG as part of their clinical care and during said cEEG had at least one seizure or instance of status epilepticus (SE) from October 2018 to October 2022 were included. For each patient, clinical and EEG variables were collected from a retrospective review of the EPIC electronic medical record. To create a balanced model reflective of the typical end of monitoring for cEEG patients, each patient had their last seizure and second-to-last seizure (if # of seizures ≥ 2) as the starting point for time-to-event analysis. A survival function was developed based on these variables to allow for a robust and appropriate representation of patients at the end of their EEG monitoring. In total, 187 time-to-event intervals were analyzed. As seizures can often cluster, we required at least a 1-h gap between seizures to start a new time-to-event data point, which we have termed a “seizure block”.

Clinical variables: Clinical data were obtained retrospectively from the EPIC electronic medical record. Variables were grouped into four categories: demographics and history (age, sex, prior acute clinical seizures, epilepsy, and status epilepticus); admission characteristics (type of brain injury: ischemic stroke, cardiac arrest, traumatic brain injury, encephalopathy, neoplasm, subarachnoid hemorrhage, subdural hematoma, or intracranial hemorrhage and Confusion Assessment Method and Richmond Agitation-Sedation Scale scores at admission); medications (number and type of anti-seizure medications and intravenous anesthetics administered); and seizures on EEG (total number of seizures during hospitalization, maximum seizure burden within a single hour of EEG, mean seizure burden, and duration of the most extended continuous period with more than one seizure per hour).

### EEG

After patients with seizures were identified, EEGs were manually reviewed to mark seizure onsets and seizure endpoints as defined by the absence of epileptiform patterns. Additional electrographic features were also assessed, such as lateralized periodic discharges (LPDs), lateralized rhythmic delta activity (LRDAs), generalized rhythmic delta activity (GRDAs), generalized periodic discharges (GPDs), Brief (Ictal) Rhythmic Discharges (BRD), bilateral independent periodic discharges (BIPDs), and sporadic epileptiform discharges (SED). Each EEG was also read to identify background features, including primary background frequency, presence of sleep features, posterior dominant rhythm, focal slowing, and burst suppression. SE was defined as a seizure lasting longer than 10 min or more than 20% of any given hour containing seizures.^15^ cEEG monitoring length was determined by the attending physician per clinical protocol. Two board-certified clinical neurophysiologists confirmed all EEG findings.

### Statistical analysis

A univariate time-to-event analysis was conducted using Cox regression using the survival Package in R [17]. The primary outcome was time to seizure recurrence or EEG termination, with all intervals censored at 72 h to minimize informative censoring. Differences in seizure-free survival were assessed using log-rank tests, and Kaplan–Meier curves were generated to visualize the survival distributions.

The proportional hazards assumption was verified by confirming that survival curves did not cross on graphical inspection. Categorical variables were compared using Fisher’s exact test, while continuous variables were compared using means and non-parametric methods (Wilcoxon). Statistical significance was defined as two-sided *P* < 0.05. All analysis was performed in R. Since only one variable was significant in the univariate analysis, multivariate analysis was not conducted due to insufficient statistical power for comprehensive analysis. 95% confidence intervals were developed using bootstrapping.

## Results

**Fig. 1:**
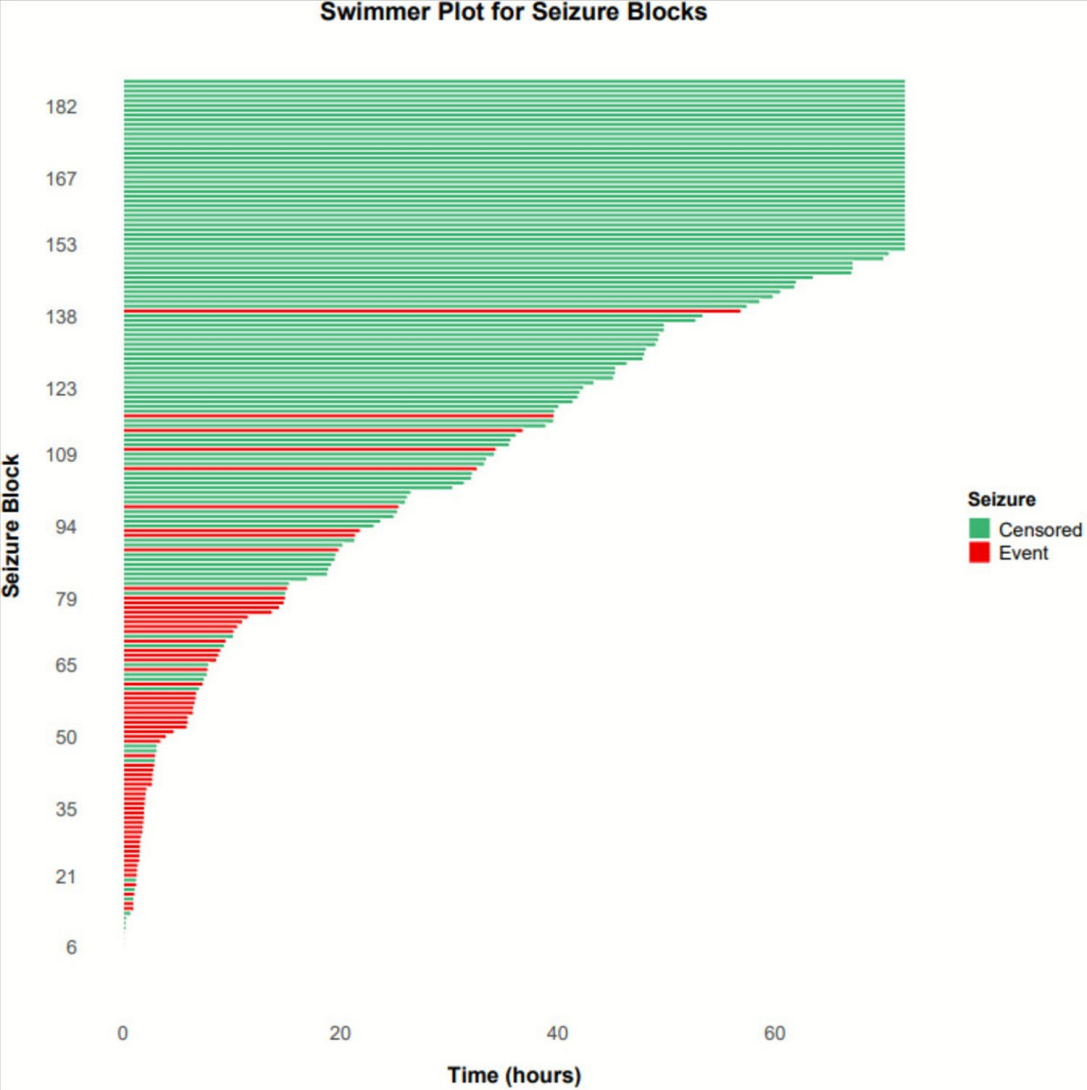
Swimmers plot showing the duration of EEG monitoring for the 187 seizure blocks on the 117 patients. Green are patients who did not have a seizure recurrence (shows duration of EEG prior to termination–censored), red represents when a seizure recurred

A total of 117 patients who experienced electrographic or electroclinical seizures were included in the analysis. The mean age was 58.4 years (SD 16.4), and 56.4% (*n* = 66) were female. The average EEG monitoring duration was 40.5 h (SD 26.4), with a mean of 22.8 total seizures recorded. Patients experienced a median of 1.6 seizure blocks during monitoring. Clinical characteristics of the cohort are summarized in Table 1. Notably, 40.2% of patients had a documented history of SE, 36.8% had a history of epilepsy, and 74.4% presented with altered mental status (AMS). Coma on admission was observed in 41.9% of patients, while sporadic epileptiform discharges (SED) were present in 46.2%. Generalized EEG slowing was observed in 79.5% of cases, and 36.8% of patients received intravenous anesthetics during monitoring. Swimmers plot for seizure blocks showing the duration of monitoring prior to seizure recurrence (red) versus cessation of EEG (green) is presented in Fig. 1.

**Table 1:** Baseline clinical and EEG characteristics (*N* = 117)

Category	Variable	Mean (SD)
Demographics/monitoring	Age, years	58.4 (16.4)
	Female sex, %	56.4
	EEG duration, h	40.5 (26.4)
Seizure burden	Total seizure number	22.8 (57.8)
	Seizure blocks per subject, *n*	1.6
Clinical presentation	Status epilepticus, %	40.2
	Acute clinical seizure, %	71.8
	Coma,%	41.9
	Altered mental status (AMS), %	75.2
	Alert, %	24.8
	Modified Rankin Scale at admission	1.7 (1.1)
	Glasgow Coma Scale at admission	8.3 (5.4)
Etiology/history	History of epilepsy, %	36.8
	lschemic stroke, %	5.6
	Traumatic brain injury, %	7.7
	Non-traumatic cerebral hemorrhage, %	12.8
	CNS tumor, %	10.3
	Liver/kidney disease or toxic ingestion, %	4.3
	Other systemic disease, %	28.2
Treatment	Number of anti-seizure medications	2.6 (1.4)
	IV anesthetics, %	36.8
EEG findings	LPDs, %	22.2
	GPDs, %	7.7
	LRDA,%	4.3
	BIPDs, %	1.7
	BRDs/B(l)RDs %	11.1
	Sporadic epileptiform discharges (SEO), %	47.0
	Generalized slowing, %	79.5
	Focal slowing, %	43.6
	Burst-suppression, %	12.8

**Fig. 2:**
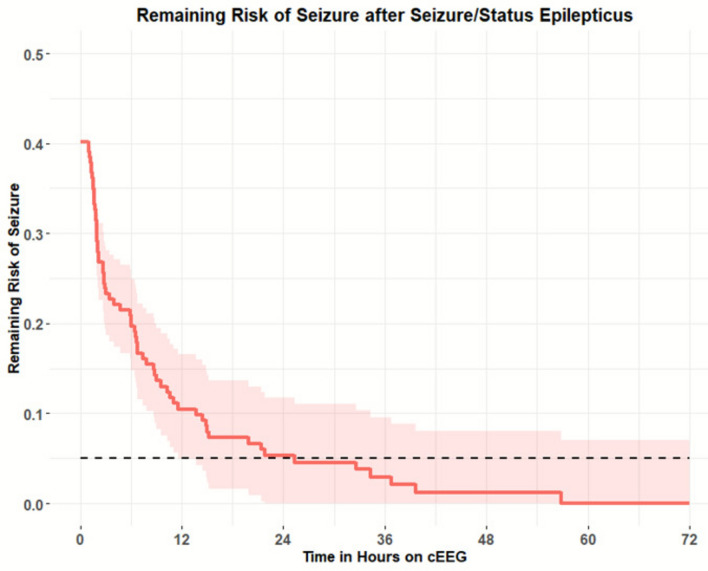
Survival analysis plot with remaining risk of seizure recurrence. Presented with 95% confidence intervals. Dashed line represents the threshold at 5% seizure risk (25.5 h)

**Fig. 3:**
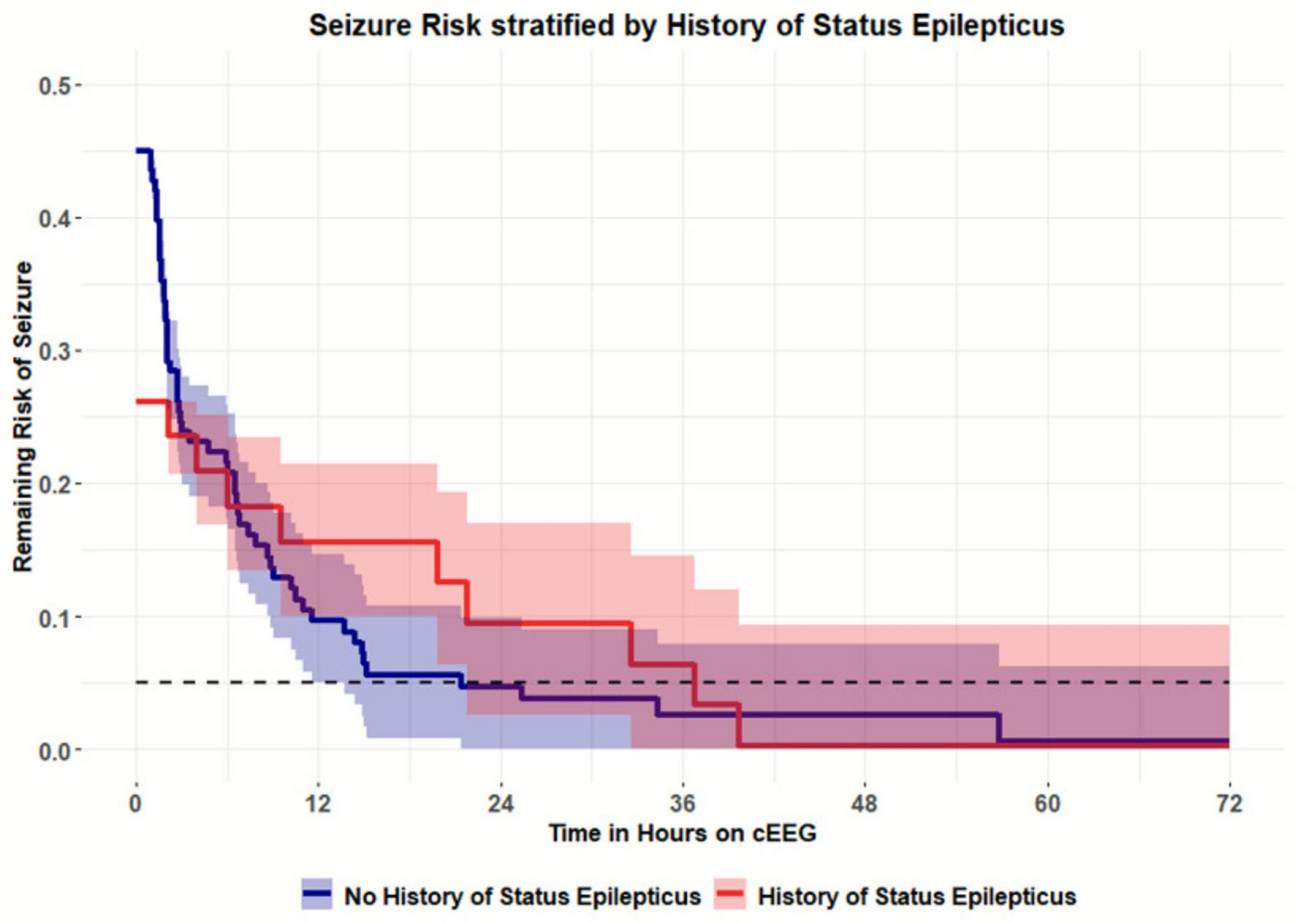
Survival analysis plot with remaining risk of seizure recurrence stratified by prior status epilepticus on EEG. Presented with 95% confidence intervals. Blue is no history of status epilepticus and red is with history of status epilepticus. Dashed line represents the threshold at 5% seizure risk, which is crossed at 36.8 h with status epilepticus and 21.2 h with no history of status epilepticus

Univariate time-to-event analysis was performed on 187 seizure-free intervals to assess the association between clinical features and the risk of seizure recurrence during EEG monitoring. Among the variables tested, only a history of SE was significantly associated with increased seizure recurrence risk (*P* = 0.022) (Table 2). For all patients, the duration of EEG needed for a < 5% chance of seizure was 25.5 h, presented in Fig. 2 with 95% confidence intervals. Patients with SE had a lower probability of remaining seizure-free compared to those without SE, as illustrated in the Kaplan–Meier survival curves (Fig. 3). The estimated duration of EEG monitoring required to reduce seizure recurrence risk to below 5% was 36.8 h in patients with SE, compared to 21.2 h in those without SE (Table 3).

**Table 2:** Univariate analysis (*N* = 117)

Category	Variable	*N* (%)	Mean (SD)	*P*-value
Demographics	Age		58.4 (16.4)	0.838
	Sex: female	66 (56.41)		0.365
	Sex: male	51 (43.59)		
Monitoring/seizure burden	EEG duration, h		40.5 (26.4)	
	Total seizure number		22.8 (57.8)	0.185
	Seizure blocks per subject		1.6	
Clinical presentation	Status epilepticus	47 (40.17)		**0.022**
	Acute clinical seizure	84 (71.79)		0.501
	Coma	49 (41.88)		0.155
	AMS	87 (74.36)		0.275
	Alert	29 (24.79)		0.282
Etiology/history	History of epilepsy	43 (36.75)		0.069
	lschemic stroke	6 (5.13)		0.545
	Traumatic brain injury	9 (7.69)		0.344
	Non-traumatic hemorrhage	14 (11.97)		0.670
	CNS tumor	12 (10.26)		0.548
	Toxic-metabolic encephalopathy	5 (4.27)		0.943
	Other systemic disease	32 (27.35)		0.998
Treatment	ASM added after seizures			0.547
	Number of ASMs		2.6 (1.4)	0.441
	IV anesthetics	43 (36.75)		0.418
EEG findings	LPDs	25 (21.37)		0.965
	GPDs	9 (7.69)		0.387
	LRDA	5 (4.27)		0.669
	BIPDs	1 (0.85)		0.995
	BRDs/B(l)RDs	12 (10.26)		0.975
	SEO	54 (46.15)		0.101
	Generalized slowing	93 (79.49)		0.692
	Focal slowing	51 (43.59)		0.343
	Burst-suppression	14 (11.97)		0.761

Bold value indiicates statistically significant result

**Table 3:** Duration of EEG monitoring for < 5% risk of seizure recurrence

Variable	Coefficient	*P*-value	Duration EEG for < 5% risk of seizure (h)
All subjects			25.25
History of status epilepticus (SE)	2.293	**0.022**	36.8
No history of status epilepticus			21.2

Other clinical variables, including coma, history of epilepsy, and the presence of sporadic epileptiform discharges, use of anesthetic infusion, and addition of an anti-seizure medication, did not reach statistical significance. The 72-h seizure risk for patients with and without these features ranged from 35 to 45%, but none met the threshold for inclusion as significant predictors, so multivariate models were not attempted.

## Discussion

This time-to-event analysis of 117 patients, encompassing 187 seizure blocks, found that the average EEG monitoring duration required to reduce seizure recurrence risk below 5% ranged from approximately 21.2 h for patients without status epilepticus up to 36.8 h for those with a history of status epilepticus. This finding aligns with prior studies and current practice recommendations of at least 24–48 h of cEEG monitoring after seizure occurrence [3, 18–21]. Notably, patients with a history of status epilepticus (SE) demonstrated a significantly higher risk of seizure recurrence during monitoring (*P* = 0.022), indicating SE as a key predictor of elevated seizure risk. This suggests that while SE signals a period of acute seizure vulnerability, its associated risk resolves in a relatively predictable timeframe. In contrast, other clinical factors, such as a history of epilepsy or sporadic epileptiform discharges, did not reach statistical significance.

Our results highlight the importance of status epilepticus (SE) as a key clinical variable associated with early seizure recurrence during EEG monitoring. Patients with a history of SE exhibited a significantly elevated seizure risk within the first 72 h, suggesting that this group benefits from more prolonged EEG surveillance. These findings are consistent with prior studies. For instance, across selected ICU cohorts undergoing cEEG monitoring, reported rates of NCSE range from 8 to 48%, reflecting its high prevalence in critically ill populations [7, 22–25].

The most important confounding variable is the use of anesthetic infusions. These agents suppress ictal activity and confound the duration of EEG required to ensure a low rate of seizure risk recurrence. ~ 37% of patients were on anesthetic-related infusions. The general practice is to wean these medications and monitor for seizure recurrence [18, 23, 26–28]. Their use was not associated with a difference in seizure recurrence. This likely reflects real-world practice, since these agents are used mainly for status epilepticus, and assessment of recurrence risk is deferred until after infusions are discontinued and the patient is observed off sedation.

Our study encounters several limitations. First, although a range of clinical features were analyzed, the use of univariate survival models limits the ability to control for potential confounding between variables. Some clinical predictors that showed trends toward significance, such as coma, history of epilepsy, and sporadic epileptiform discharges, will require larger sample sizes to allow for multivariate modeling to assess their impact fully [11, 20]. A more detailed investigation of anesthetic infusion rates and agents used, such as pentobarbital, requires longer times to clear [13, 29, 30]. Second, the analysis was restricted to a 72-h monitoring window to minimize censoring bias, which may have excluded late-occurring seizures [31]. Lastly, the retrospective nature of the study and single-center design may introduce selection bias and limit broader applicability [11, 14]. Future prospective multicenter studies are warranted to validate these findings and explore integrated risk prediction strategies.

This study reinforces the value of continuous EEG monitoring in seizure risk assessment, particularly in patients with a history of status epilepticus. Our time-to-event analysis demonstrated that SE is significantly associated with seizure recurrence, and longer than 24 h should be considered, particularly during infusion weaning. These findings support the clinical practice of extended EEG monitoring following seizures, especially in high-risk populations. Continued research is needed to develop refined, patient-centered strategies for seizure detection and management in critically ill patients.

## Data Availability

The data that support the findings of this study are available from the corresponding author upon reasonable request. Data sharing is subject to institutional approvals and privacy restrictions.

## Abbreviations

AMS: Altered mental status
ASM: Antiseizure medication
BIPD: Bilateral independent periodic discharges
BRD: Brief rhythmic discharges
cEEG: Continuous electroencephalography
EEG: Electroencephalography
GPD: Generalized periodic discharges
ICU: Intensive care unit
KM: Kaplan–Meier
LPD: Lateralized periodic discharges
LRDA: Lateralized rhythmic delta activity
NCSE: Nonconvulsive status epilepticus
SED: Sporadic epileptiform discharges
SE: Status epilepticus
